# High Photon Absorptivity of Quantum Dot Infrared Photodetectors Achieved by the Surface Plasmon Effect of Metal Nanohole Array

**DOI:** 10.1186/s11671-020-03326-9

**Published:** 2020-05-05

**Authors:** Hongmei Liu, Yongqiang Kang, Tianhua Meng, Cuifeng Tian, Guodong Wei

**Affiliations:** 1 Institute of Solid State Physics, Shanxi Datong University, Datong City, 037009 People’s Republic of China; 2 School of Physical Science and Electronics, Shanxi Datong University, Datong City, 037009 People’s Republic of China; 3 Key Laboratory of Physics and Technology for Advanced Batteries (Ministry of Education), Jilin University, Changchun City, 130012 People’s Republic of China

**Keywords:** Quantum dot, Surface plasmon, Infrared photodetector, QDIP, Nanohole array

## Abstract

With the increasing demand for small-scale photodetector devices, quantum dot–based infrared photodetectors have attracted more and more attention in the past decades. In this work, periodic metal nanohole array structures are introduced to the quantum dot infrared photodetectors to enhance the photon absorptivity performance via the surface plasmon enhancement effect in order to overcome the bottleneck of low optical absorption efficiency that exists in conventional photodetectors. The results demonstrate that the optimized metal nanohole array structures can greatly enhance the photon absorptivity up to 86.47% in the specific photodetectors, which is 1.89 times than that of conventional photodetectors without the metal array structures. The large enhancement of the absorptivity can be attributed to the local coupling surface plasmon effect caused by the metal nanohole array structures. It is believed that the study can provide certain theoretical guidance for high-performance nanoscale quantum dot–based infrared photodetectors.

## Background

Semiconductor infrared photodetectors can be used to detect the infrared light and have great application promising in the fields of scientific research, digital imaging, optical communication, and military areas. To date, the quantum dot infrared photodetectors (QDIPs) have attracted increasing attention in recent years due to their outstanding light response properties and trends toward device miniaturization [1–3]. Though decades of sustained efforts scientists have made great progress in developing technologies for obtaining high-performance QDIPs, it still needs to make further improvements to meet the challenges of device miniaturization [4] and practical demands. It is pointed out that coupling metal grating on the quantum dot–based active region could be regarded as an effective approach to enhance the performances of the QDIPs [5, 6], which can yield high photoabsorption coefficient by the local photocoupling of plasmon-enhanced effect.

According to reports, there are two main kinds of metal grating structures used to improve the performance of the QDIPs. One is the metal hole array structure, and the other is the no-hole metal array structure. More concretely, in the application respect of the metal hole array structure, Chang’s group combined the metal hole periodic-array with the quantum dot layer in the QDIP in 2007, which led to the supernormal light transmission of the photodetector [7]. In 2009, Lee et al. proposed a method for high-detectivity QDIP by integrating metallic photonic crystals with a 3.6-μm period of hole array (100-nm thickness) [8]. The research results demonstrate that the method can realize the peak response of the photodetector at the wavelength of 11.3 μm and yield up to 30 times enhanced detectivity than that without the metal photonic crystal. Then, they discussed the performance of QDIP dependence on the incident light and their application in the focal plane array [9, 10]. A similar performance enhancement can be also found in the reports by using the hole metal array coupling grating [11–14]. No-hole metal array structures are also proposed. In 2011, Huang and his coworkers used the self-organized plasmon silver nanoparticle layer to enhance the wide spectral response of the QDIP and obtained 2.4~3.3 times enhancement [15]. In 2014, Chen’s group reported that the performance of the photodetector can be enhanced through the near-field effect of the Au nanoparticles [16]. In 2015, Ding’s group and Wang’s group proposed the waveguide coupling structures and the single resonant cavity of distributed Prague reflector [17, 18], respectively. Besides the abovementioned structures, other metal structures, for example, the antenna strips array and the nano-disk array were also discussed and analyzed [19–21].

However, these no-hole array methods can also exhibit enhanced effect in the photo-response of the QDIPs, but its fabrication process with cost-effective and simple methods still remains a challenge compared with the typical hole array structure. For the typical hole array structures, the size of the hole array is common at the microscale. The photo-response enhancement is through the plasmon effect at the interface between the air in the microscale metal holes and the semiconductor below. The metal hole array size can be further decreased to expected nanoscale matching to the size of the quantum dot in the nanoscale QDIP, whether the high-performance QDIPs with corresponding enhancement effect can be realized or not. At the same time, further theoretical works are necessary to elucidate the mechanisms underlying these phenomena. In this study, to make the phenomenon clearly, the QDIPs with the nanoscale metal hole array structures are designed, and more important compared with the conventional microscale QDIPs, the enhancement effect is explained by analyzing the optical transmission condition and the distribution of the electric field. The results demonstrate that the QDIPs with the nanoscale metal array structures can have the photon absorptivity up to 86.47% due to the photon-quantum dot interaction and the efficient light coupling, which may open the door to the design and the optimization of the nanosize infrared photodetector.

## Design Model of the QDIP with the Nanohole Array

**Fig. 1:**
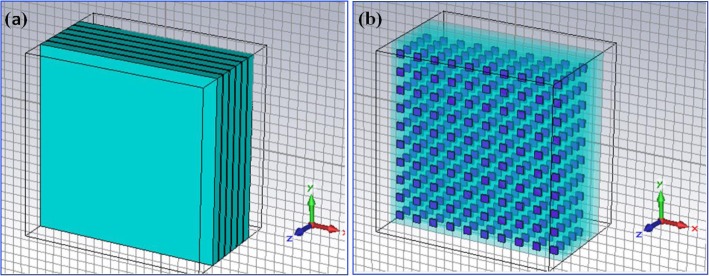
**a** Structural model diagram of the typical quantum dot infrared photodetector. **b** Quantum dot distribution in the active regions with 5-period quantum dot composite layers. These composite layers are constituted by the A1GaAs barrier layer and the GaAs layers

**Fig. 2:**
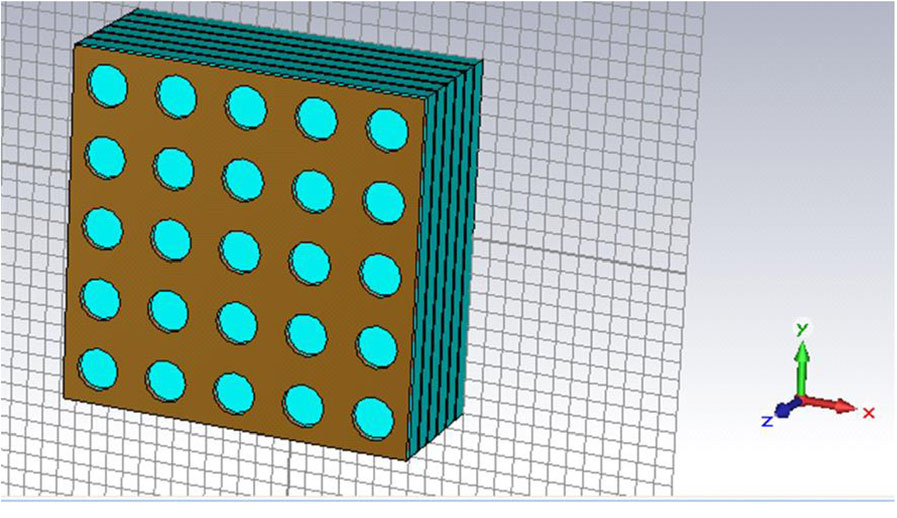
Quantum dots active regions with the periodic metal nanohole array structures for the improved QDIP

**Fig. 3:**
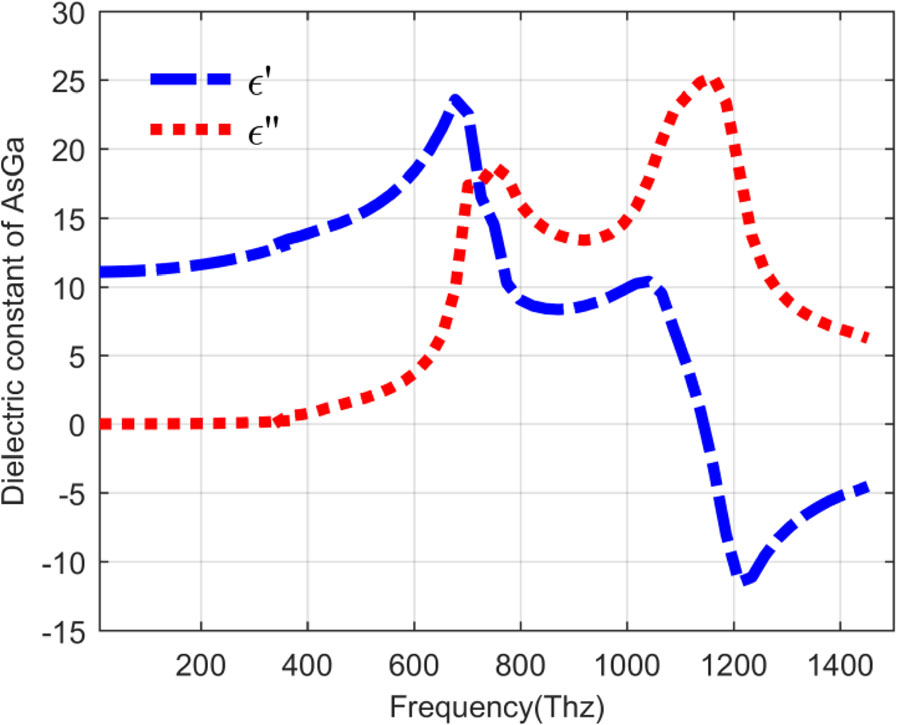
Dispersion relation of GaAs material

**Fig. 4:**
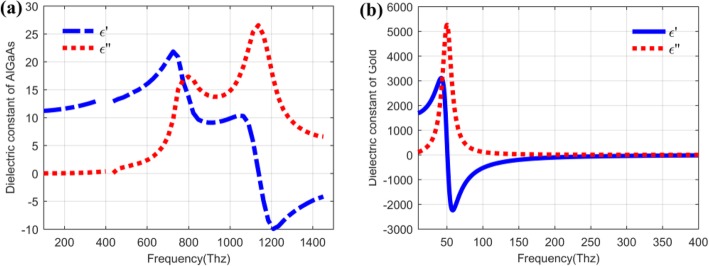
**a** Dispersion relation of AlGaAs material. **b** Dispersion relation of Au material

In general, the QDIP consists of the quantum dot region and the electrodes, and the quantum dot region is made up of period quantum dot layer and barrier layers. Under ideal conditions (ignoring the influences of the electrodes and the substrate), the optical transmission of the whole QDIP can be supposed to be equal to that of the quantum dot region. Thus, the electrodes and the substrate are not needed to appear in the design of the QDIP. Specifically, Fig. 1 a gives the design of the typical QDIP, which is made up of 5-period quantum dot composite layers, and these composite layers are constituted by the A1GaAs barrier layer and the GaAs layers including the periodic quantum dots (Fig. 1b). In the current configuration, the quantum dot nanoparticles are supposed as the cube shape which is in line with the definition of quantum dots formed with many atoms and the molecules, and it is 40 nm in length, 40 nm in width, and 7~9 nm in height. A similar model of quantum dots can be also found in the reported literature [22]. The area of the QDIP is set as 1000 nm × 1000 nm, and the thickness of the AlGaAs barrier layers is 60 nm. The metal nanohole array chosen as Au is placed on the conventional quantum dots nanostructure layers of the conventional QDIP, which is named as the improved QDIP displayed in Fig. 2. The radius of the holes can be adjusted in the range of 50~70 nm. It is noted that the material used to make up the quantum dot cannot be simply regarded as a bulk material with a certain refractive index. Figure 3 reveals the electric dispersion characteristic of the GaAs material used to form the quantum dot by using the method of Edward D. Palik [23]. In the figure, the blue curve and the red curve represent the dielectric constant of GaAs *ε*^′^and *ε*^″^, respectively. Figure 4 a and b show the electric dispersion characteristic of the GaAs, Al_0.3_Ga_0.7_As material and gold material, respectively.

## Calculation Method Based on the YEE Cell

Based on the above physical model designed, the method of the finite element integration is used to calculate the condition of the optical transmission. First of all, based on the YEE cells, the Maxwell integral equations satisfying the model above are written as follows:

1 $$ {\oint}_LE\cdot dl=-\frac{\partial }{\partial t}{\int}_SB\cdot dS $$

2 $$ {\oint}_LH\cdot dl=J+\frac{\partial }{\partial t}{\int}_SD\cdot dS $$

3 $$ {\oint}_SD\cdot dS=q $$

4 $$ {\oint}_SB\cdot dS=0 $$

**Fig. 5:**
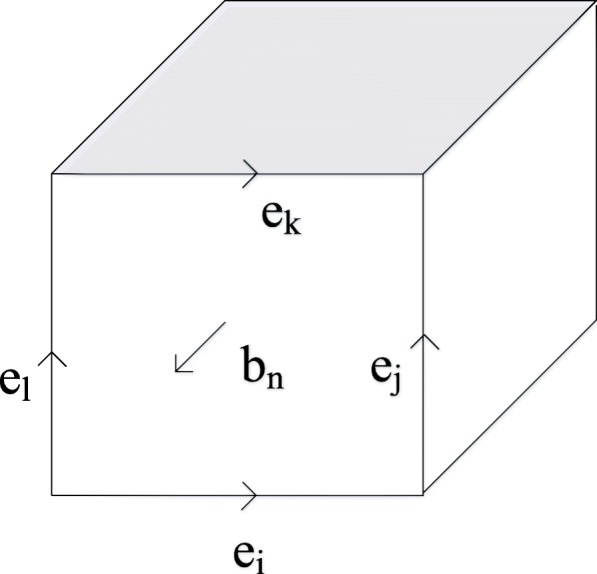
Schematic diagram of the “YEE cell”

In our calculation, Eqs. 1–4 are discretized. Both the distribution of the electric field nodes and the magnetic field nodes are chosen as “Yee cell” format. Using Eq. 1 as an example, the electromagnetic model of the photodetector can be taken as the accumulation of “Yee cell”. As shown in Fig. 5, the four sides of the arbitrary cell corresponds to Eq. 1, representing the electrical-field vector *e*_*i*_,*e*_*j*_,*e*_*k*_, and *e*_*l*_. The vector located in the normal direction is the magnetic field vector *b*_*n*_, and thus the previous Eq. 1 can be rewritten as the following Eq. 5.

5 $$ {e}_i+{e}_j-{e}_k-{e}_l=-\frac{db_n}{dt} $$

Adopting a similar method, the equation of the electromagnetic model of the whole photodetector can be written as:

6 $$ \left[\begin{array}{l}..\dots \dots \dots \dots \dots \dots \dots \\ {}1\kern0.5em 1\kern0.5em -1\kern0.5em -1\\ {}.\dots \dots \dots \dots \dots \dots \dots \\ {}.\dots \dots \dots \dots \dots \dots \dots \end{array}\right]\left[\begin{array}{l}{e}_i\\ {}{e}_j\\ {}{e}_k\\ {}{e}_l\end{array}\right]=-\frac{d}{dt}\left[\begin{array}{l}.\\ {}{b}_n\\ {}.\\ {}.\end{array}\right] $$

Equation 2 can be also rewritten as:

7 $$ Ce=-\overset{.}{b} $$

According to the similar method, the other *Eqs.*2 ~ 4 can be discretized as:

8 $$ \overset{\sim }{C}h=\overset{.}{d}+j $$

9 $$ sb=0 $$

10 $$ \overset{\sim }{S}d=q $$

Combining the discretized Eqs. 7–10 with the bounding condition, the electric field and the magnetic field can be solved by the iterative method. In this study, the metal hole array structure is placed on the top of the conventional QDIP, and as such, the structure can facilitate light coupling through the Bragg scattering. The corresponding optical communication is further calculated, which can be discussed in the following section when the condition of the transmission, the reflection, and the absorption is given. Furthermore, based on the relationship between the absorption and the quantum efficiency, the responsivity of the QDIP can be given. To be concreted, as well known that the responsivity of the QDIP as a very important performance parameter can be calculated by the ratio of the photocurrent and the power of the incident light [24]. So, it can be written as:

11 $$ R=\frac{I_{photo}}{P_o}=g\frac{\eta e}{hv} $$

where *I*_*photo*_ is the photocurrent of the QDIP, *P* is the power of the incident light, *g* is the photoconductive gain, *e* is the charge of the electron, *h*is the Planck constant, *v* is the frequency of the incident light, and *η* is the quantum efficiency.

The quantum efficiency can be defined as the ratio of the number of the electron hole and that of the incident photo, which strongly depends on the absorption of the photodetector. In practice, since the incident light directly illuminates the absorption region, it cannot be absorbed completely due to the reflection of the top contact layer or the metal layer [25, 26]. Thus the quantum efficiency of the QDIP can be written as:

12 $$ \eta =\left(1-r\right)\left[\exp \left(-{\alpha}_0d\right)\right]\left[1-\exp \left(-{\alpha}_0W\right)\right] $$

where *α*_0_*W* is the absorbtion coefficient of the QDIP, *α*_0_*d* is the absorbtion coefficient of the incident contact layer, *r* is the reflection of the incident layer, respectively.

In the QDIP, the photoconductive gain can be defined as the ratio of the recombination time of electrons from an extended state back into a quantum dot *τ*_*life*_ to the transit time of electrons across the device *τ*_*total*_, and it can be shown as:

13 $$ g=\frac{\tau_{life}}{\tau_{total}} $$

and under the condition that the transit time across one period of the quantum dot composite layer is considerably smaller than the recombination time from an extended state back into a quantum dot [22, 27]_,_ the gain can be written as:

14 $$ g=\frac{\left(K+1\right) L\mu E{\left[1+{\left(\mu E/{v}_s\right)}^2\right]}^{\hbox{-} 1/2}}{\mathrm{K}\pi {a}_{QD}^2{h}_{QD}^2{\sum}_{QD}{V}_{\mathrm{t}}} $$

where *K* is the number is the quantum dot composite layer, *L* is the distance between quantum dot layers, *μ* the mobility of electrons, *E* is the electric field density across the QDIP, *v*_*s*_ is the saturation velocity of electrons, *h*_*QD*_ is the height of the quantum dots, ∑_*QD*_ is the quantum dot density in each quantum dot layer, *a*_*QD*_ is the lateral size of quantum dots, and *V*_t_ is the capture rate of electrons, respectively.

Submitted Eq. (12) and Eqs. (14) into Eq. (11), we can obtain the responsivity of the QDIP, which can be shown as :

15 $$ R==\frac{\lambda \left(K+1\right) L\mu E{\left[1+{\left(\mu E/{v}_s\right)}^2\right]}^{\hbox{-} 1/2}\left(1-r\right)\exp \left(-{\alpha}_0d\right)\left[1-\exp \left(-{\alpha}_0W\right)\right]}{1.24\mathrm{K}\pi {a}_{QD}^2{h}_{QD}^2{\sum}_{QD}{V}_{\mathrm{t}}} $$

## Results and Discussion

**Fig. 6:**
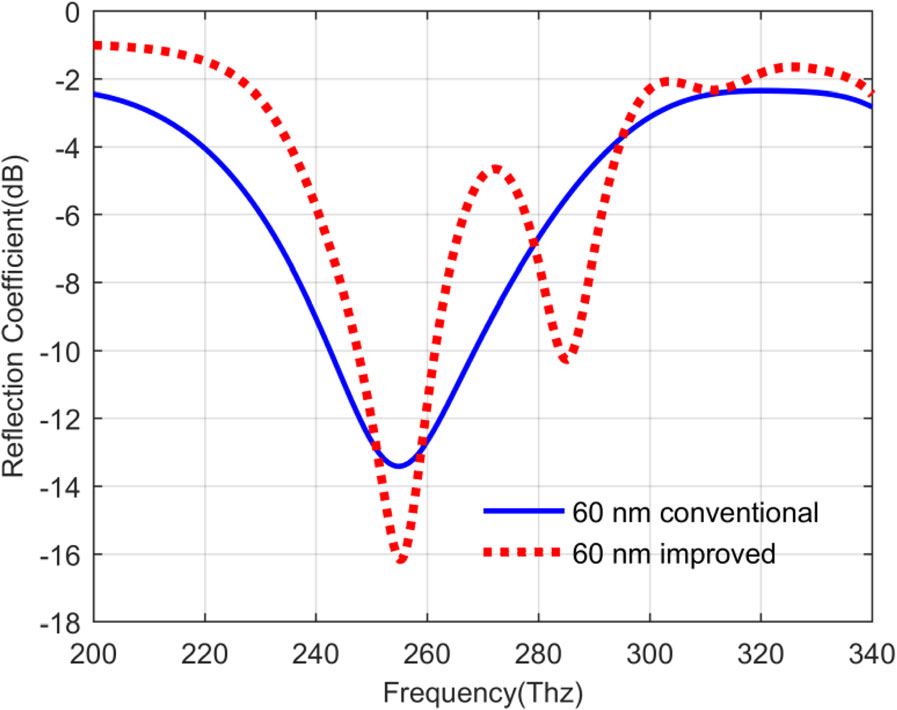
Reflection coefficients of the conventional QDIP without metal array (blue curve) and the improved QDIP with the metal nanohole array (red curve)

**Fig. 7:**
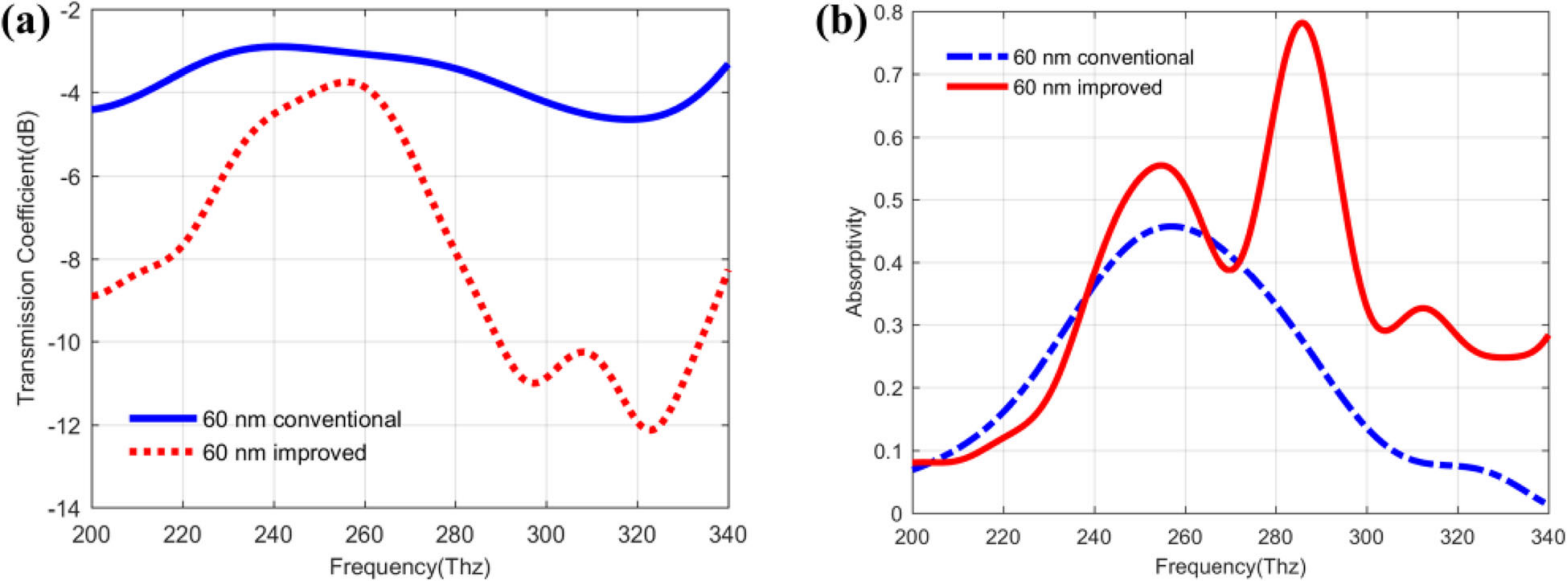
Transmission coefficients (**a**) and absorptivity coefficients (**b**) of the conventional QDIP and the improved QDIP, respectively

Based on the design of the QDIP above, if the incident infrared light strikes on the top of these QDIPs in the *z*-axis direction, the photodetectors will have the reflection and the transmission of the incident light. The absorptivity of the photodetectors can be determined by studying these optical transmission conditions of the incident light, which can play a very important role in evaluating the performance of the photodetector. Figure 6 presents their reflection conditions of the photodetector without the metal array (the conventional QDIP) and that with the metal array (the improved QDIP). Compared with the two curves in Fig. 6, it can be found that the reflection coefficient values of the conventional QDIP are lightly smaller than that of the improved QDIP besides the individual values in the frequency ranges of 250~260 Thz and 279~293 Thz. Specifically, taking the value at the frequency of 219 Thz as an example, the reflection coefficient value of the conventional QDIP is -3.91 dB, while the improved QDIP is as low as − 1.31 dB. As what has been said above, the improved QDIP can have a slightly higher value than the conventional QDIP, but it can be found that the minimum absorption of the improved QDIP is quite smaller than that of the conventional QDIP. To be specific, the minimum absorption of the improved QDIP is − 16.17 dB at the frequency of 255.10 Thz, whereas the value for the conventional QDIP is equal to − 13.42 dB at 254.86 Thz. The low reflection coefficient of the improved QDIP could be attributed to the higher absorption coefficient of metal than that in the semiconductor for infrared light. The absorption can be calculated out based on the common contributions of the reflection and the transmission. Figure 7 a depicts the transmission coefficient of the conventional QDIP and their values marked with the blue color are clearly bigger than those of the improved QDIP within the total frequency range of 200~340 Thz. For instance, at the frequency of 298 Thz, the transmission coefficient of the improved QDIP is only − 10.83 dB, which is 1.60 times smaller than that of the conventional QDIP, which is − 4.15 dB. According to the competitive relationship among the transmission, the reflection and the absorption, the decrease of the transmission coefficient will lead to the increase of the absorption under the condition of ignoring other losses of the incident light.

Combined the transmission situation in Fig. 7a with the reflection situation in Fig. 6, the absorptivity of the QDIP can be calculated, which is shown in Fig. 7b. In the figure, the red curve (marked as 60 nm improved) describes the absorptivity of the improved QDIP with the metal structure, and the other blue curve represents the conventional QDIP without the metal hole structure (marked as 60 nm conventional). Making a comparison between the two curves, it can be found that the absorptivity of the improved QDIP is higher than that of the conventional QDIP. The maximum of the absorptivity of the improved QDIP is 0.782 at the frequency of 286 Thz, which is 1.71 times higher than that of the conventional QDIP, which is only 0.458 at the frequency of 257 Thz. The reasons for the increase in the absorptivity of the improved QDIP can be explained as follow. The metal nanohole array structures are introduced onto the top of the conventional QDIP, and such configuration can favor the surface plasmon resonance effect, leading to the local coupling effect of the incident light. Furthermore, the local coupling effect can make more incident light enter into the semiconductor quantum dot layers below, which can result in high absorption for the incident light and have better photoelectric properties with larger photocurrent and higher quantum efficiency.

**Fig. 8:**
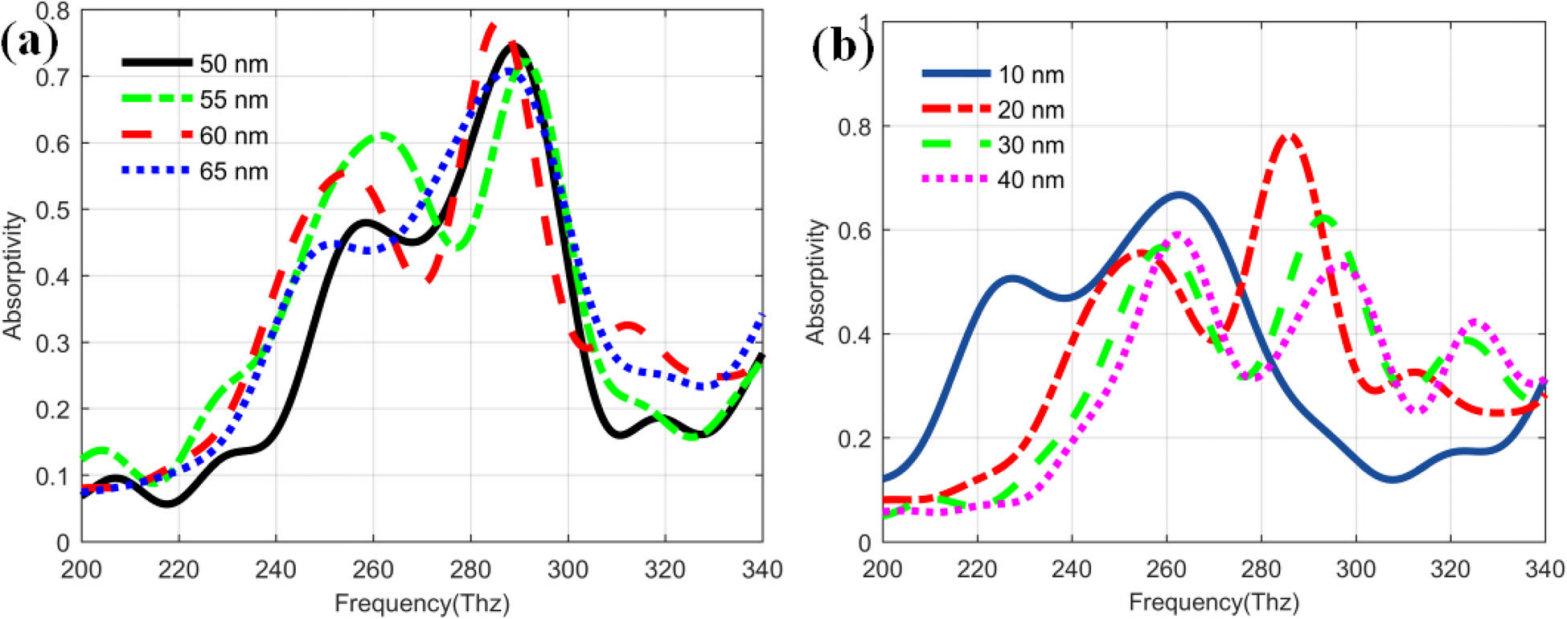
Absorptivity of the improved QDIP (**a**) with different radiuses and different metal thicknesses of metal nanoholes (**b)**

To further make clear that how to realize the plasmon enhancement effect in the improved QDIPs, we also study the influences of the different metal nanohole structures on the absorptivity of the improved QDIPs. As shown in Fig. 8a, the absorptivity curves of the improved QDIPs with different metal nanohole radius correspond to the black (50 nm), green (55 nm), red (60 nm), and blue (65 nm) curve, respectively. The absorptivity values of the improved QDIP reveal the different changing trend under different nanohole. The peak values of the absorptivity for the improved QDIP is 0.744 (black curve at 289 Thz), 0.721 (green curve at 291 Thz), 0.782 (red curve at 286 Thz), and 0.707 (blue curve at 288 Thz), respectively. Obviously, among these photodetectors, the improved QDIP with a hole radius of 60 nm can have the best absorptivity performance. At the same time, it is well known that the thickness of the metal hole layer can also have the influences of the absorptivity. As demonstrated in Fig. 8b, when the thickness of the metal layer in the improved QDIP is changed from 10 to 40 nm, the peak values of the absorptivity correspondingly changes from 0.667 (263 Thz for the 10-nm thickness) to 0.782 (286 Thz for 20 nm), 0.662 (293 Thz for 30 nm), and 0.590 (262 Thz for 40 nm). Among these peak values, the metal nanohole layer with the thickness of 20 nm can have the highest absorptivity value.

**Fig. 9:**
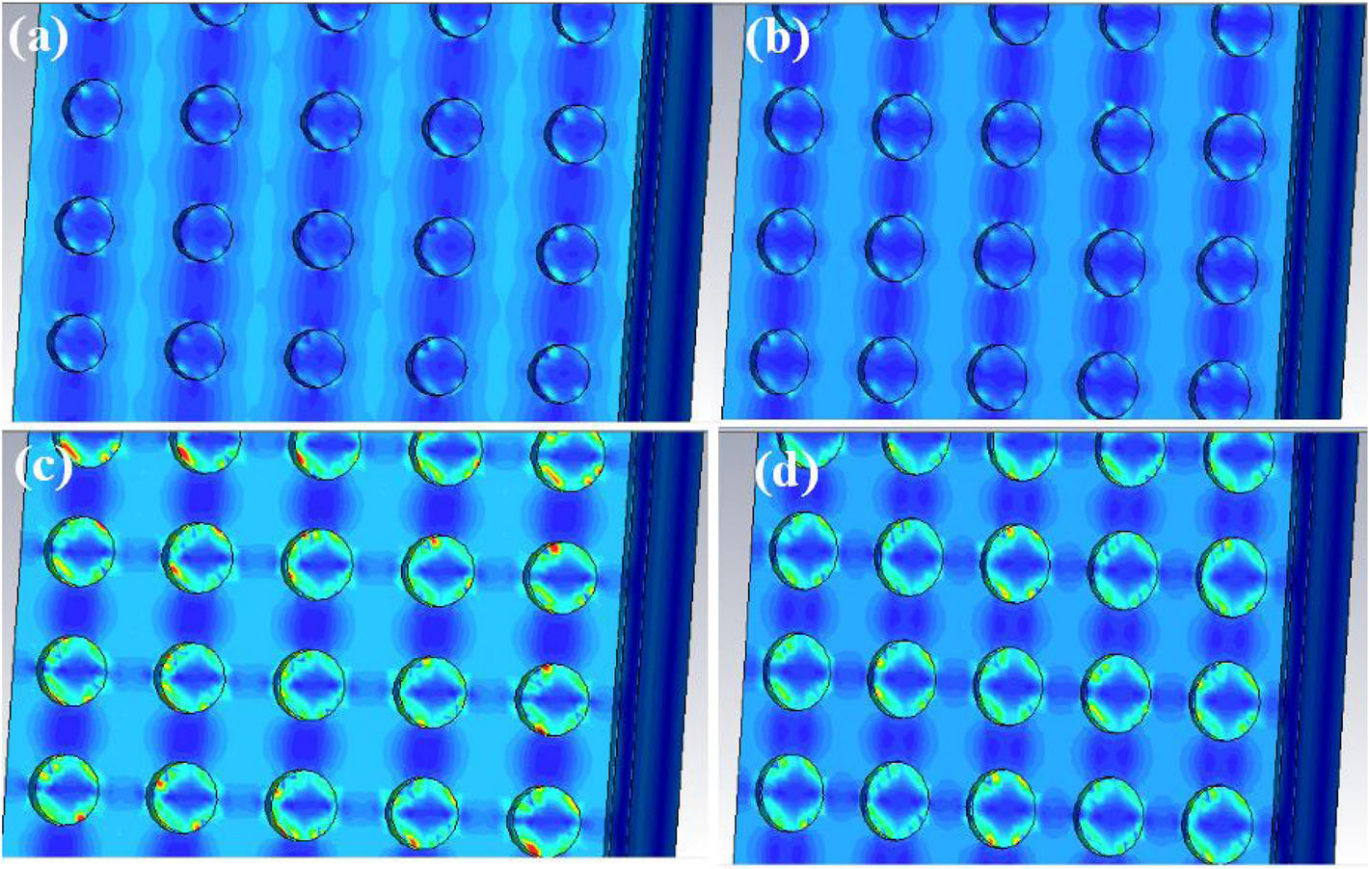
Electric field distribution of the improved QDIPs with different radiuses of metal nanoholes: **a***r* = 50 nm, **b***r* = 55 nm, **c***r* = 60 nm, and **d***r* = 65 nm

**Fig. 10:**
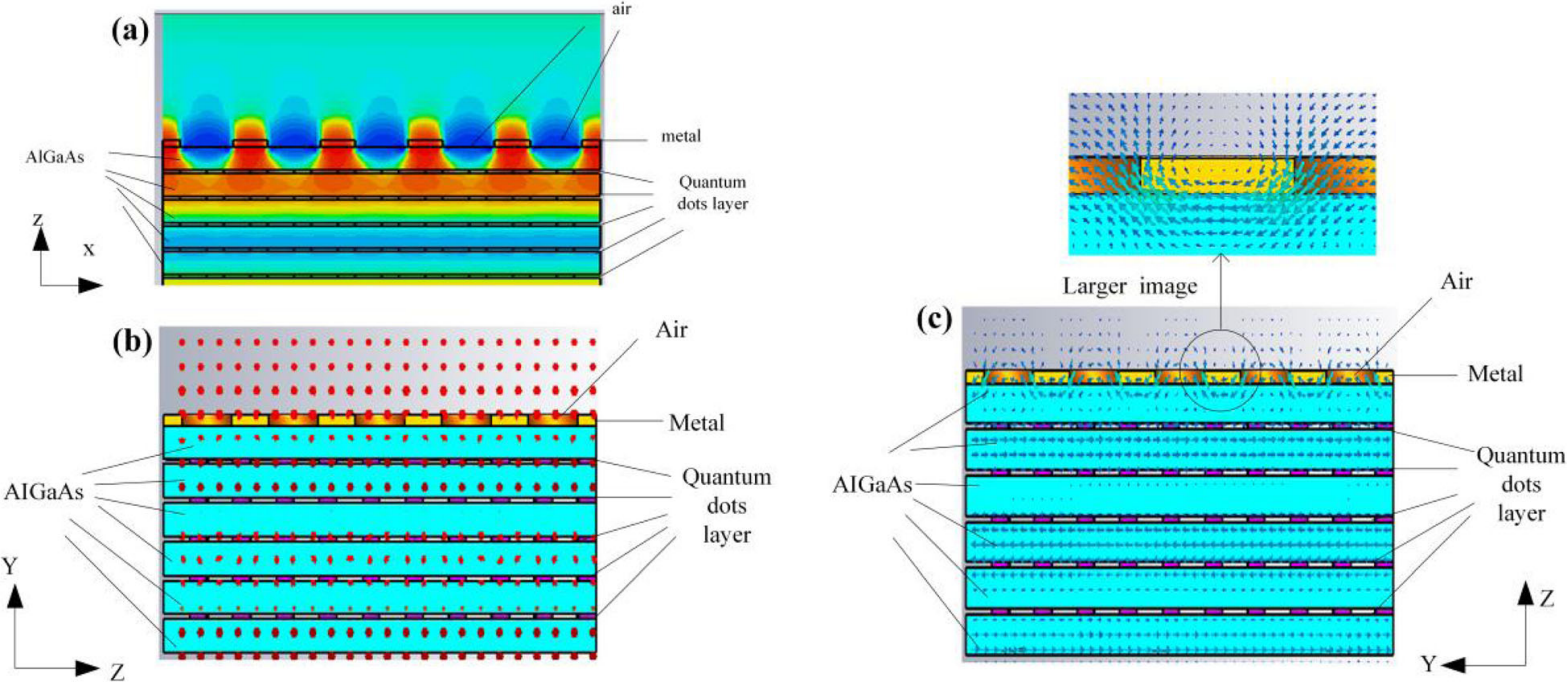
**a***x*-direction elecrtic field of the optimized QDIP. **b** Electric field polarization of the improved QDIP. **c** Magnetic field polarization of the improved QDIP

To make the above phenomenon clear, we further study the distribution conditions of the electric field at the top surface of the improved QDIP with the different radiuses of the metal holes at 286 Thz. Figure 9 reveals the electric field distribution at different radiuses of metal holes in the range of 50–65 nm. Compared the four pictures in Fig. 9a, it is clear that the QDIPs with the hole radius of 50 nm (Fig. 9a) and 55 nm (Fig. 9b) can have relatively weaker electric field enhancement due to the corresponding electric field distributions in Fig. 9 a and b lying on the whole area including the holes and their adjacent regions, and thus their electric field local coupling effects can be ignored, whereas the local coupling area of the electric field with a high electric field can be observed in Fig. 9 c and d. The strong electric field distributions around the holes in Fig. 9c and d with the shape of the ring can be located on the interface between the metal holes and the air in the metal holes as a result of the surface plasmon coupling effect. Compared with the electric field distributions in Fig. 9c and d, the electric field coupling effect in Fig. 9c is stronger than that in Fig. 9d according to their marked colors which are a mixture of red, green, and blue. In this regard, the red color is on behalf of the strongest field, and the blue color represents the weakest field. Based on the above analysis, the metal nanoholes with the radius of 60 nm do generate the enhancement electric field effect by the surface plasmon. To further make the enhancement effect more clear, the distribution of the electric field on *xz*-plane corresponding to the maximum absorption of the optimized QDIP at the frequency of 286 Thz in our study as shown in Fig. 10a, which lies on the section *y* = 0 (corresponding to the field of the *xz*-plane). In the figure, from the direction of *z*-axis, the enhanced electric field distribution lies in the region between adjacent metal holes which is marked with the red color and the weak field lies in the region of the metal holes marked with the blue color. The electric field distribution directly reveals the enhanced absorption of the QDIP. It is the enhancement coupling effect that leads to the increase of the absorptivity, and further leads to the high quantum efficiency of the improved QDIP. Of course, the same conclusions can be also drawn by analyzing the magnetic field distribution according to the properties of the electromagnetic properties of light. Due to the discussion of the magnetic field distribution is the same as that of the electric field distribution, it is no need to discuss in this study.

In addition, as what has been said above, the enhancement effect is from the surface plasmon, and the mode of the excited surface plasmon wave is further determined. Figure 10a and b display the electric and magnetic field results. In the figure, Fig. 10b exhibits the polarization distribution of the electric field on *yz*-plane. It can be seen that the electric field is normal to the *yz*-plane, that is to say, the electric field cannot have the *E*_z_ component. Figure 10c presents the polarization distribution of the magnetic field. It can be found that the magnetic field is parallel to the *yz*-plane, that is to say, there is the Hz component in the propagation direction of the incident light which is *z*-direction. Hence, in our study, the excited surface plasmon wave is the TE mode. Furthermore, to make clear the position used to excite the surface plasmon, the distribution of the magnetic field near the metal hole interface is shown on the top of Fig. 10c. It can be seen the magnetic field on the position between adjacent metal holes is stronger than that in the metal holes. In addition, according to the electric field appeared in Fig. 10a, it can also prove that the enhancement field is concentrated on the location between adjacent metal holes. Therefore the conclusion can be drawn that the surface plasmon effect can be from the surface between metal and semiconductor, which lies on the position between adjacent metal holes. Of course, it is worth noting that the enhancement of the absorption is not only from the surface plasmon but also from the enhanced reflection of the metal layer, leading to the secondary absorption of the incident light since the incident light is illuminated on the QDIP along z-axis.

**Fig. 11:**
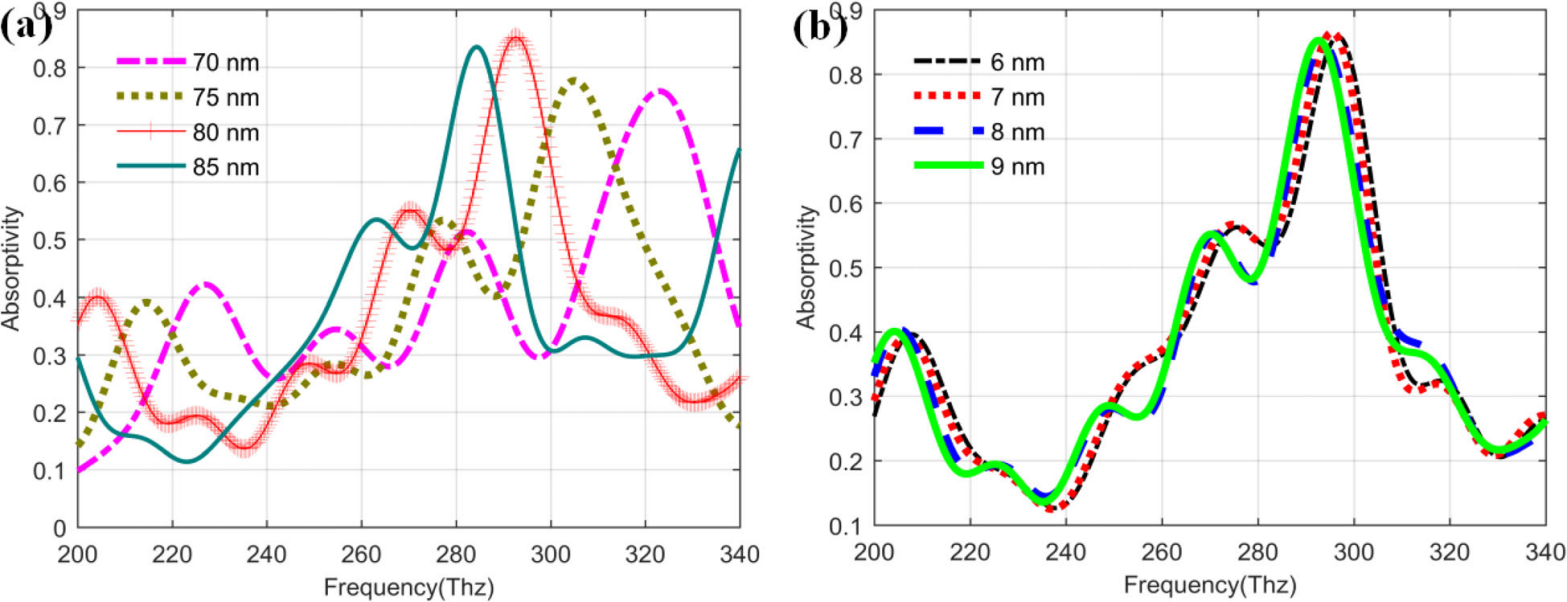
Absorptivity of the improved QDIP with the different thicknesses of **a** the quantum dot layer and **b** barrier layer

It is well known that the parameters related to the metal layers can have also great influences on the performance of the QDIP. In order to determine the optimal parameters, the thickness of the barrier layer and the quantum dots layer are further analyzed and discussed under the conditions of the optimized metal layer thickness (20 nm) and metal hole radius (60 nm). Figure 11a depicts the changing trend of the absorptivity of the photodetectors with the different thicknesses of the barrier layer in the range of 70–85 nm. From the image, these absorptivity curves have the similar change trend. When the thicknesses of the barrier layer vary in the range of 70~85 nm, the corresponding maximum absorptivity values of the improved QDIPs are 0.7581 (70 nm, at 322.78 Thz), 0.7763 (75 nm, at 304.84 Thz ), 0.8552 (80 nm, at 292.75 Thz), and 0.8346 (85 nm, at 284.17), respectively. Compared with these maximum absorptivity values, it can be found that the barrier layer with the thickness of 80 nm can have the best absorptivity performance for the improved QDIPs. Fixed other parameters with the above optimized values, the influences of the thicknesses of the quantum dot layer on the absorptivity performance for the improved QDIP are further studied, and the corresponding curves are shown in Fig. 11b. From the figure, it can be found that the red curve can have the maximum absorptivity value of 0.8647 at the frequency of 295.48 Thz for the improved QDIP with the layer thickness of 7 nm, which illustrates that the photodetector can have the optimal transition state.

**Fig. 12:**
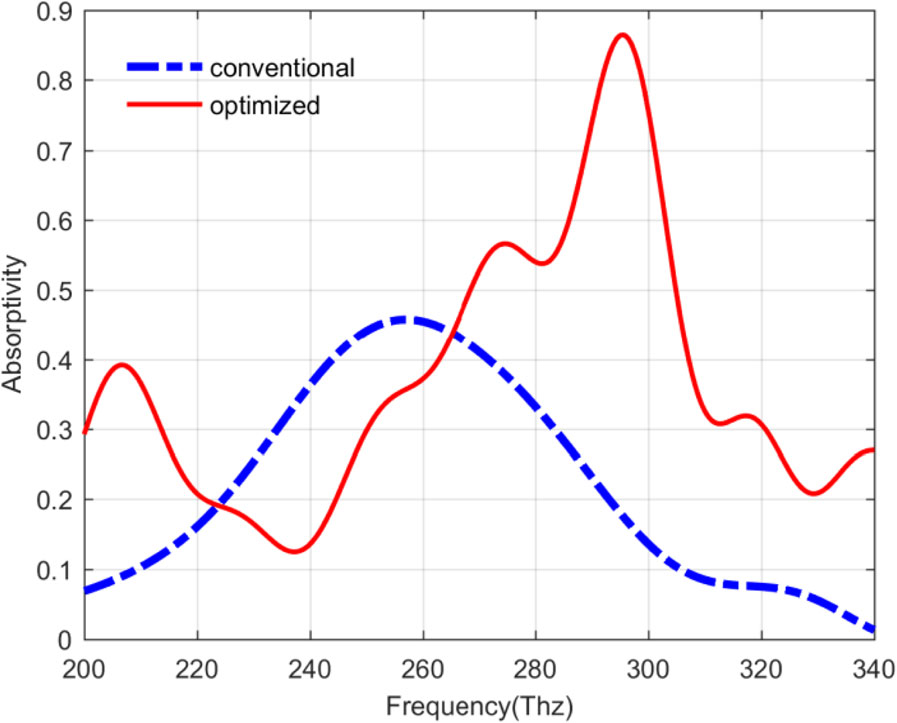
Absorptivity of the conventional QDIP in blue curve and improved QDIP in red curve with the barrier layer thickness of 80 nm

Based on the above discussion, it is clear that not only the parameters related to the QDIP can have the influences on the device performance, but also the thicknesses of the quantum dot layer and the barrier layer can also determine device performance. In this study, according to the theoretical calculation results, the optimized parameters for the improved QDIPs can be given with the metal layer thickness of 20 nm, the metal hole radius of 60 nm, the quantum dots layer thickness of 7 nm and the barrier layer thickness of 80 nm. The absorptivity of the optimal photodetector can be as high as 0.8647. Moreover, comparing the conventional QDIP with the optimized QDIP as shown in Fig. 12, the absorptivity values in the red curve are quite higher than that in the blue curve besides the values in the frequency range of 222.91~262.18 Thz. The maximum absorptivity in the red curve is equal to 0.8647 at the frequency of 295.48 Thz, which is 1.89 times bigger than that in the blue curve (which is the same as the previous curve marked “60 nm conventional” in Fig. 7b corresponding to the QDIP without metal hole array) at the frequency of 257 Thz. The frequency shift for the maximum absorptivity peak mainly results from the change in the thickness of the improved photodetector. Furthermore, based on the optimized parameters of the QDIPs, the thicknesses of the quantum dot layer and the barrier layer, the quantum efficiency value and the responsivity of the photodetector are calculated out.

### Quantum Efficiency and Responsivity of the QDIP

**Fig. 13:**
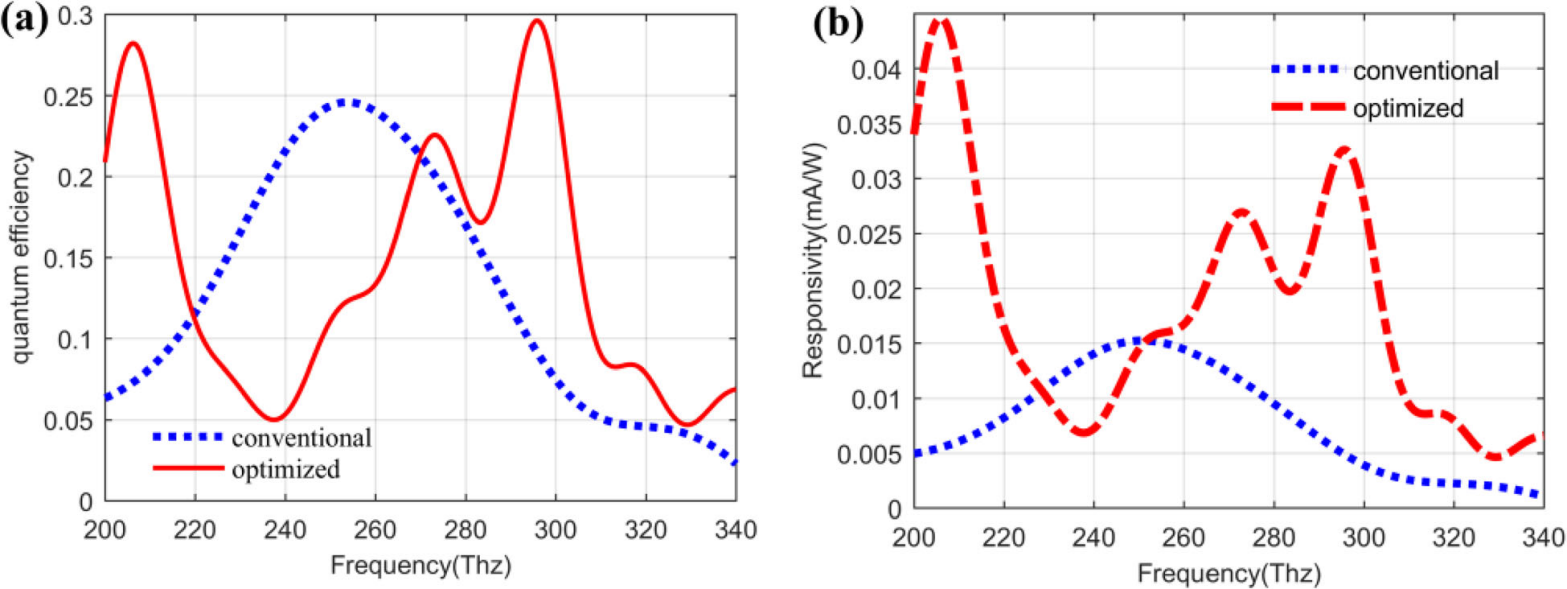
**a** Quantum efficiency of QDIP. **b** Responsivity of QDIP

Based on the calculated results of the absorptivity in Fig. 12 as well as combining with the expression of the quantum efficiency and the responsivity of the QDIP above, the quantum efficiency of the QDIP and the responsivity can be calculated out, and the corresponding results are plotted in Fig. 13 a and b. Figure 13a depicts the quantum efficiency of the QDIP. In this figure, the blue dotted curve represents the quantum efficiency of the QDIP without metal array, the other red full curve is that of the optimized QDIP with metal array. Making a comparison between the two curves, it can be observed that the maximum quantum efficiency of the optimized QDIP is 0.2961 at the frequency of 295.87 Thz, and it is 1.205 times than that of the conventional QDIP, which is equal to 0.2458 at the frequency of 256.48 Thz. The increasing trend is similar to the absorptivity provided in Fig. 12 which results from the introduction of the metal hole array and the optimization of the quantum dot infrared photodetector. Based on the increasing trends for the absorptivity, we can find that the responsivity of the QDIP also reveals similar increasing trends. To be specific, Fig. 13b gives the responsivity of the optimized QDIP and the conventional QDIP, respectively. In the figure, the red curve is on behalf of the responsivity of the conventional QDIP, and the blue curve stands for that of the optimized QDIP with the metal holes layer. Similar to the analysis in Fig. 13a, the responsivity is 0.0326 mA/W at the frequency of 295.87 Thz, which is 0.0174 larger than that of the conventional QDIP at the frequency of 256.48 Thz (which is 0.0152). The increase in the responsivity can be proven in the other frequency band in the range of 229.57~254.41 Thz, which obviously demonstrates the enhancement in the performance of the photodetector due to the introduction of the metal hole array and the optimization of the quantum dot regions. Moreover, the reasons for the enhancement were detailedly discussed in detail analyzing the electric field distribution of quantum dot regions above.

### Influence of the Electrodes and the Substrate

**Fig. 14:**
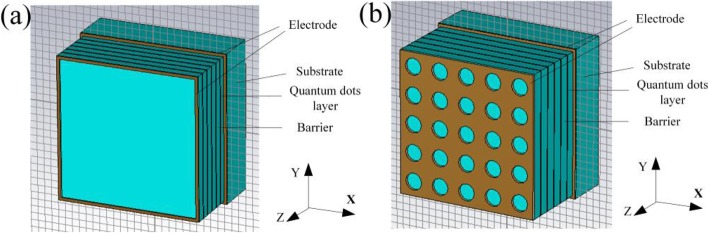
**a** Conventional QDIP with electrodes and substrate. **b** Optimized QDIP with the electrodes and substrate

What was studied above is all under the condition of ignoring the influence of the electrodes and the substrate; in fact, the electrodes and the substrate can have certain influences on the performance, but they do not influence the description of the enhancement effects of the performance of the optimized QDIP with the metal layer. This is because the electrodes and the substrate can have almost the same influence on the absorption of the QDIP with the metal layer and without the metal layer. To illustrate the issue adequately, we recalculated the absorption of the QDIP considering the influence resulting from the substrate and the electrodes as well as the quantum efficiency, the responsivity, and so on. To be concrete, it is well known that the electrodes are generally designed at the two ends of the absorption region of the quantum dots, and thus, as shown in Fig. 14a, one is at the top of the conventional QDIP and the other is at the bottom end of the absorption region of the QDIP. That is to say, it lies at the top of the substrate, which can provide the quantum dot absorption region with the bias voltage and transmit current together with the electrodes said above. Here, it is worth mentioning that there is the metal hole array instead of the metal ring in the optimized QDIP in our study used as the electrodes. The other electrode is similar to that of conventional QDIP. Based on the above design, a concrete distribution of the electrodes is clearly calculated in Fig. 14a. Similar to Fig. 14a, in Fig. 14 b, the material of the electrodes is chosen as gold, and the substrate is chosen as AlGaAs; their thicknesses are 20 nm and 300 nm, respectively.

**Fig. 15:**
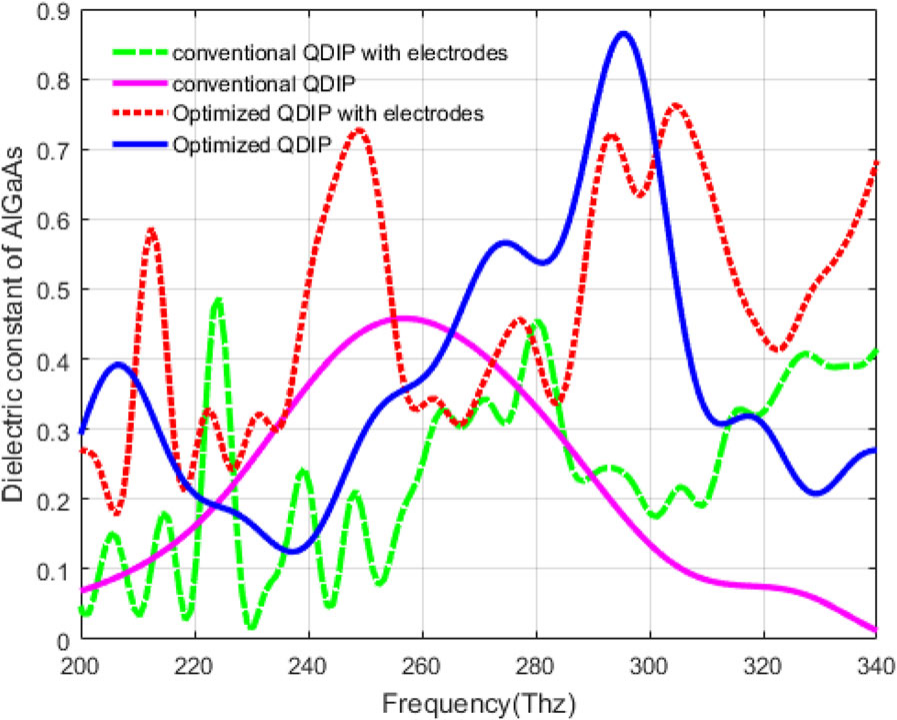
Absorption of the QDIP with electrodes and substrate

Based on the introduction of electrodes and substrate in the structure of the QDIP above, we calculated the absorption of the QDIPs, and the corresponding results are compared with the QDIP without the electrodes and the substrate. Figure 15 demonstrates the influence of the electrodes and substrate on the absorptivity of the QDIP. In the figure, the blue curve and the pink curve are the absorptivity of the conventional QDIP without the electrodes and the substrate and that of the optimized QDIP without the electrodes and the substrate, respectively. The red dashed curve represents the absorptivity of the optimized QDIP with the electrodes and the substrate. Its maximum absorption is 0.7620 at the frequency of 304.35 Thz which is just 0.1027 smaller than that of the optimized QDIP without electrodes and substrate. The decrease of the absorptivity is degraded from the loss of the electrodes and the substrate, the same as the green curve with the absorptivity of the conventional QDIP with the electrodes and substrate. Compared with the absorptivity of the conventional QDIP and optimized QDIP with electrodes and substrate, the enhancement is very clear in the absorptivity of the optimized QDIP with electrodes and substrate, which is the same as the optimized QDIP in Fig. 12. In other words, though the electrodes and the substrate can result in the decrease of the absorptivity, the total absorptivity of the optimized QDIP can be enhanced compared with that of bare QDIP, and thus, the decrease can be negligible as they can have a very small influence on the description of the enhancement of the optimized QDIP when using the metal hole array.

In addition, it can be observed that the change trend of the absorptivity of the QDIP with the electrodes and substrate cannot be the same as the previous curves (corresponding to the blue curve and pink curve). They are more complex with many peak values. The reasons for this phenomenon can be explained as follows. First of all, the addition of the electrodes and the substrate can produce more or less a loss and the frequency shifts due to the accumulated heating effect and the other negative influence factors. Secondly, since the material of the electrodes is chosen as the metal, in the optimized QDIP with the electrodes and the substrate, it can result in enhanced reflection and enhanced surface plasmon. The two reasons commonly favor the enhancement of the absorptivity as demonstrated in Fig. 15.

## Conclusions

In conclusion, the conventional QDIP performance can be greatly improved by adding the nanoscale metal nanohole array, and the enhanced mechanism of the performance for improved QDIPs is discussed by analyzing the reflection, the transmission, the absorption, and the distribution of the electric field. The results not only demonstrate that the improved QDIPs can have higher absorptivity than that of conventional QDIPs but also indicate that the parameters of the improved QDIPs related to the metal nanohole array together with the quantum dot composite layer can significantly influence their performance. According to theoretical calculation, the optimized parameters of the improved photodetectors are 20 nm in metal layer thickness, 60 nm in metal hole radius, 7 nm in quantum dot layer thickness, and 80 nm in barrier layer thickness. The maximum absorptivity value of the optimized photodetector can be as high as 86.47% at the frequency of ~ 300 Thz. The great enhancement of the absorptivity can be attributed to the local coupling effect caused by the enhancement of the electric field effect via the surface plasmon, and further leads to the high quantum efficiency and responsivity, which are 0.2961 and 0.0326 mA/W, respectively. It is believed that the current contribution could provide certain theoretical guidance for developing nanoscale QDIPs with high performance.

## Data Availability

All data are fully available without restriction.

## Abbreviations

QDIPs: Quantum dot infrared photodetectors Fig Figure Eqs Equations
